# Availability and affordability of children essential medicines in health facilities of southern nations, nationalities, and people region, Ethiopia: key determinants for access

**DOI:** 10.1186/s12889-021-10745-5

**Published:** 2021-04-13

**Authors:** Tefera Tadesse, Habtamu Abuye, Gizachew Tilahun

**Affiliations:** 1 Pharmacy Unit, Pharmaceutical Supply Management, Doctor Bogalech Gebre Memorial General Hospital, Durame, Ethiopia; 2 Department of Pharmacy, College of Medicine and Health Sciences, Wachemo University, P.O. BOX: 667, Hossaena, Ethiopia; 3 Pharmacoepidemiology and Social Pharmacy Department, School of Pharmacy, Institute of Health Sciences, Jimma University, Jimma, Ethiopia

**Keywords:** Essential medicine, Children, Availability, Affordability, Price, SNNPR, Ethiopia

## Abstract

**Background:**

Children in resource-limited countries are more likely to die from treatable conditions than those in higher resource settings due to a lack of the right essential medicine at the right time. Globally millions of children die every year from conditions that could be treatable with existing medicines before they reach their fifth birthday. This study aimed in assessing the availability and affordability of essential medicine for children in selected health facilities of southern nations, nationalities, and peoples’ regions (SNNPR), Ethiopia.

**Method:**

A medicine outlets-based cross-sectional study was conducted to assess the availability, affordability, and prices of the 30 selected essential medicines (EMs) for children in 30 public and 30 private medicine outlets in SNNPR from March 29 to May 5, 2019, applying WHO and Health Action International (HAI) tools. Availability was expressed as the percentage of sampled medicine outlets per sector that the surveyed medicine was found on the day of data collection. The amount of daily wages required for the lowest-paid government unskilled worker (LPGW) to buy one standard treatment of an acute condition or treatment for a chronic condition for a month was used to measure affordability and median price ratio for the price of EMs.

**The results:**

Availability varied by sector, type of medication, and level of health facilities. The average availability of EM was 57.67% for the public sector and 53.67% for the private sector. Ceftriaxone, SOR, zinc sulfate, and cotrimoxazole were the most widely available types of medications in the two sectors. The median price ratios (MPR) for the cheapest drugs LP were 1.26 and 2.24 times higher than their International Reference Price (IRP) in the public and private sectors respectively. Eighty-two percent of LP medicines in the public and 91 % of LP medicines in the private sectors used in the treatments of prevalent common conditions in the region were unaffordable as they cost a day’s or more wages for the LPGW.

**Conclusion:**

Availability, affordability, and price are determinant pre-requisite for EMs access. According to the current work, although fair availability was achieved, the observed high price affected affordability and hence access to EMs.

**Supplementary Information:**

The online version contains supplementary material available at 10.1186/s12889-021-10745-5.

## Background

A high standard of health is a basic right for every human [1]. Individuals and societies have the responsibility to ensure that this basic human right is achieved. Access to essential medicines (EMs) is a necessary tool for ensuring the health of individuals and communities. EMs have been identified in prior research based on community health relevance, evidence on efficacy, safety, and comparative cost-effectiveness [2]. EMs are expected to be available from health systems at all times in adequate amounts, in the proper dosage forms, with assured quality and sufficient information, and at a price, the individual and the society can afford [3].

However, access to EMs is challenging; especially for children. Some of the factors which impaired children’s access to EMs were lack of suitable dosage forms, the high price of medicines, inefficient government procurement culture, extreme mark-ups in the distribution chain, and exaggerated taxes and duties being applied to these medicines [4–7]. Even though its necessity was emphasized in Millennium Development Goals/MDG/ four and six, Sustainable Development Goals (SDG) goal 3 and WHO launched the ‘Make Medicines Child Size’ campaign to enhance the availability of safe, effective, and quality medicines for children by promoting awareness and action through research, regulatory measures, and changes in policy, effective results for it has not yet been achieved [6, 8–10].

Thus, millions of children die every day before they reach their fifth birthday, of conditions that could be treatable with existing EMs globally. Of newborn deaths, 22% are due to infections such as pneumonia, diarrhea, and malaria. Childhood pneumonia and diarrhea are the most important causes of childhood mortality and account for about 30% of all child deaths worldwide [11, 12]. The majority of these children would endure if they have given appropriate available EMs [13]. For instance, oral antibiotics administered in community settings can reduce all sources of neonatal mortality by 25% and pneumonia-related mortality by 42%; zinc administration for diarrhea management can reduce all-cause mortality by 46% [14, 15]. The scenario worsens in resource-constrained nations. Children in developing countries are more liable to die from treatable conditions than those in higher resource settings due to a lack of access to the correct medication at the right time [16].

Access to EMs can be determined by availability, affordability, accessibility, acceptability, accommodation/adequacy, and/or quality of the medicines [17, 18]. However, programs such as ‘Better Children’s Medicines’ stressed that improving access to children’s EMs is more applicable by addressing issues of accessibility, safety, efficacy and price (affordability) [19]. As per the studies, assuring availability and affordability of medicines play a vital role in improving children’s access to EMs in both private and public sectors. Availability is reported as the percentage of medicine outlets in which medicine was found on the day of data collection and affordability, in other words, is estimated by comparing medicine costs to the daily wage of the lowest-paid unskilled government worker (LPGW) [6, 7].

A series of initiatives have been taken by Ethiopia to improve access to EMs. A three-tier health-delivery service system was introduced to address accessibility issue. The primary level consisting of health posts (HPs), health centers and primary hospitals are made accessible to the majority of population to provide promotion, preventive and curative services; general hospitals provide secondary level services; and specialized hospitals provide tertiary services [20]. Except HPs all public sectors have pharmacies. Regarding private sector, the pharmaceutical retail system also has three outlets levels: pharmacy (run by pharmacist), drug store (run by druggist) and rural drug vendor. Except rural drug vendor the rest can stock and dispense EMs found in the national essential drug list (NEDL) [21]. As less bureaucracy is needed and more attractive services are given, they are preferred sources of EMs.

To eliminate an interrupted drug supply, drug price variation and promote the availability, pharmaceuticals fund and supply agency (PFSA) under Proclamation No. 553/2007 based on the pharmaceuticals logistics master plan (PLMP) was established [22]. Besides, for some diseases, a relaxed program called program drugs is there to consider EMs from donors and NGOs dispense them freely [23]. Finally, to counter financial hindrance (where patients fully pay out-of pocket money for the services they get) and advance affordability, community-based health insurance (CBHI) was launched and being scaled up [24]. Further, a waiver system installed grants the poorest access to free health care and free medicines [25].

Despite these initiatives, the country is still confronted with low access to children’s EMs. In a study conducted in South-west Ethiopia, 55.65% of EMs were available, and considerable price variation among studied sectors impeded access to EMs [26]. In Western Ethiopia, the average availability of EMs for children was found to be 43%. Again the price of EMs was making treatment unaffordable, and low public awareness to participate in CBHI and the government’s weak campaign could not spare the community from paying out-of-pocket money for budget EMs [27]. This study, therefore, sought to assess the availability and affordability of CEMs based on WHO/HAI methodology to determine children’s access to EMs in Southern Ethiopia to have a semi-complete picture of the problem together with already published work [27, 28].

## Methods

### Study design, area, and period

A medicine outlets-based cross-sectional descriptive study was conducted in the SNNP region, South Ethiopia. Quantitative data was collected adapting price and availability format prepared by WHO/HAI ‘make medicine child-size project’ from March 29 to May 5, 2019 (Supplementary Annex II) [19].

### Drug outlet selection

Out of 13 administrative zones found in the region, choosing Hawasa, the capital city of the SNNP region as a center for the study, six administrative zones that can be reached within 1 day were randomly selected [19, 28]. For each district, the higher health facility (HF) in the three-tier system of the country was purposely, one primary hospital and three health centers (HCs) within 3 h travel from the higher HF were randomly selected from the lists of HFs obtained from the regional health bureau of SNNPR for the public sector since primary hospital to HC ratio was 1:12 (SNNP Regional Health Bureau) [19, 28, 29]. Similarly, lists of licensed medicine outlets for each district were obtained and closest to each public HFs (one pharmacy purposely and four drug-stores) were randomly selected for private sectors as a pharmacy to drug-store ratio was 1:15 (SNNP Regional Health Bureau). If there were no private medicine outlets found within a 10 km radius of public HF, another was considered in the near urban setting [28]. Therefore, 60 medicine outlets, 30 from private, and 30 from public sectors were considered in the current study. The chosen medicine outlets were from the different levels of HFs that are expected to stock all of the medicines included in the study.

### Selection of medicines

Twenty-three EMs were taken based on proposed formulations and strength for key tracer children medicines WHO EMLc core list as specified by the ‘Better Medicines for Children Project’ [19]. Seven medicines were added to the study list as per the prevalence and burden of diseases associated with childhood illness in the region (SNNP Regional Health bureau). For each surveyed medicine, we collected data on the lowest-priced, highest priced (instead of innovator/brand medicines), and its availability (Supplementary Annex II) [19]. But for programed medicines, which are free of charge for the public in the public sector, we checked only their availability.

### Data collection and analysis

Six data collectors were trained as per WHO/HAI methodology to do the collection task. The pre-test was undertaken in Werabe town where the trainees were trained. Being supervised and controlled for quality of data daily by Principal Investigators, 60 medicine outlets were visited to collect data on the availability and patient prices of medicines. The availability of medicine was addressed by interviewing the staff working at the facility and physically checking the study medicines for their presence as stated in the dispensing area [19]. Patient prices were taken by interviewing the staff working at the facility, reviewing the most recent price data that were recorded on the posted selling price, or referring to model 22. For data collection, the WHO/HAI standard data collection format was employed (Supplementary Annex II). For tracking the quality, processing in advance, and statistical analysis, collected data were entered into customized MS Excel from the workbook provided as part of the WHO/HAI methodology. All studied medicine outlets fulfil the WHO/HAI recommendation criteria to collect data on the selected 30 medicines (Table 1) [28].

**Table 1:** List of medicine surveyed in Southern Ethiopia

S.No.	Name of Medicine	Strength	Dosage Form	Indication
1.	Amoxicillin	125 mg/ml	Suspension	Infectious disease
2.	Amoxicillin	250 mg	Dispersible tab	Infectious disease
3.	Amoxicillin+Clavulanic acid	125 + 31.25 mg/5 ml	Suspension	Infectious disease
4.	Amoxicillin+Clavulanic acid	125 mg + 31.25 mg	Dispersible tab	Infectious disease
5.	Ampicillin	500 mg	Injection	Infectious disease
6.	Artemether +Lumefantrine	20 mg + 120 mg	Tablet	Malaria
7.	Artesunate	60 mg	Injection	Malaria
8.	Beclomethasone inhaler	100mcg/dose	Inhaler	Asthma
9.	Benzylpenicillin	1MIU	Powder	Infectious disease
10.	Carbamazepine	100 mg/5 ml	Suspension	Seizure disorder
11.	Ceftriaxone injection	1 g	Powder	Severe infection
12	Chloramphenicol injection	1 g	Powder	Infectious disease
13.	Cloxacillin	125 mg/5 ml	Suspension	Infectious disease
14.	Cotrimoxazole (Sulphamethoxazole + Trimethoprim)	200 mg + 40 mg/5 ml	Suspension	Pneumonia
15.	Diazepam l injection	5 mg/ml	Solution	Seizure disorder
16.	Ferrous salt	30 mg Fe/5 ml	Suspension	Anemia
17.	Gentamycin	40 mg/ml	Injection	Infectious disease
18.	Ibuprofen	100 mg/5 ml	Syrup	Pain/inflammation
19.	Isoniazide	100 mg	Tablet	TB
20.	Morphine	10 mg/5 ml	Oral Solution	
21.	Oral Rehydration Solution	1 litter	Powder	Dehydration
22.	Paracetamol	120 mg/5 ml	Syrup	Pain
23.	Paracetamol	125 mg	Suppository	Pain
24.	Penicillin G, Benzathine penicillin	1.2MIU	Injection	Infectious disease
25.	Phenobarbitone	30 mg	Syrup	Seizure disorder
26.	Phenytoin	50 mg	Suspension	Seizure disorder
27.	Procaine penicillin injection	1 MIU	Powder	Infectious disease
28.	Salbutamol Puff	100mcg/dose	Inhaler	Asthma
29.	Vitamin A	100,000 IU	Capsule	Xerophthalmia
30	Zinc sulfate	20 mg	Tablet	Dehydration

*MIU* Million international unit, *IU* International unit, *TB* Tuberculosis, *mcg* Micrograms

### Measuring availability and affordability of medicines

The availability of individual medicine was measured by the physical presence of them in the medicine outlets during data collections [19]. It was expressed as the percentage of sampled medicine outlets per sector that the surveyed medicine was found on the day of data collection [30]. This work applied percentage ranges: < 30% very low, 30–49% low, 50–80% fairly high, and > 80% high availability to express its findings [31].

IRP was used for comparing the prices of the 17 lowest-priced medicines [32]. Patient prices were reported as median price ratios (MPRs), which expressed as median local unit prices across health facilities divided by their median IRPs [33]. To determine whether the MPRs for patient prices are high, low, or about right, Gelders S. et al*,* work was referred. Therefore, to represent acceptable local price ratios, MPR ≤ 1.5 and MPR ≤ 2.5 cut-off points were taken for public sectors and private sectors respectively [34].

$$ MPR=\frac{Median\ Local\ Unit\ Price}{International\ Reference\ Unit\ Price} $$

The local unit price was obtained by dividing the retail price per pack by the pack size. The supplier medicine prices obtained from the MSH drug price guide 2015 were taken as the IRPs for core medicines (Supplementary Annex III) [32]. MPR was calculated by converting the median local price to United States Dollar (USD) using the exchange rate of commercial banks of Ethiopia at first data of data collection, March 29, 2019 [35].

Affordability was estimated by comparing the total price required to cover the complete course of standard treatment for prevalent diseases in the region (Supplementary Annex IV) (SNNP Regional Health bureau) with the number of daily wages of the LPGW, which was 28.57 ETB per day (0.99 USD) during data collection (Ethiopian ministry of finance and economics salary scale for the public sector) [36]. Medicines used to manage asthma (chronic condition) and six acute conditions were chosen based on WHO/HAI Standards [19, 28]. For each condition, the lowest-priced medicine costs were computed and compared. The total costs of medicine for the complete duration of therapy of acute conditions and a one-month course of chronic conditions were determined and converted to the daily wage. Then, description has given as medicines that costed less than a day’s wage to buy one standard treatment of an acute condition or treatment for a chronic condition for a month are affordable and unaffordable if they cost more [33].

## Results

### Availability of EMs

Availability was varied by type of medicine, sectors, and level of health facilities. Ceftriaxone, ORS, zink sulfate, and cotrimoxazole were available in more than 90% of medicine outlets. On the other hand, none of the sectors stocked beclomethasone inhaler, morphine 10 mg syrup, and carbamazepine 100 mg syrup while isoniazid 100 mg tablet and vitamin A capsules being stocked by public sectors. The availability of nine studied medicines was less than 50%. Public sectors hold the lowest-priced medicines, unlike private sectors which had both the lowest and highest priced medicines (see Table 2).

**Table 2:** Average availability of individual children essential medicines in the public and private sectors

Name of medicine, strength, dosage form	Percentage of outlets where medicine found		
	Public Sector (*n* = 30)	Private Sector (*n* = 30)	
	LP	LP	HP
Amoxicillin 250 mg Dispersible tablet	53.33	13.33	0
Amoxicillin 125 mg/5 ml Suspension	86.67	93.3	6.67
Amoxicillin + Clavulinc acid 125 mg + 31.25 mg Dispersible tablet	6.67	6.67	0
Amoxicillin + Clavulanic acid 125 mg + 31.25 mg/5 ml Suspension	66.67	86.67	20
Ampicillin 500 mg Powder for Injection	73.33	70	0
Artemether + Lumefantrine 20 mg + 120 mg Dispersible Tab	76.67	83.33	0
Artesunate 60 mg powder for Injection	30	10	0
Benzylpenicillin 1 MIU Powder for Injection	56.67	26.67	0
Beclomethasone 100mcg/dose inhaler	0	0	0
Carbamazepine 100 mg/5 ml Suspension	0	0	0
Ceftriaxone 1 g Powder for Injection	90	100	23.33
Chloramphenicol 1 g Powder for Injection	13.33	6.67	0
Cloxacillin 125 mg/5 ml Suspension	66.67	60	0
Cotrimoxazole (Sulphamethoxazole + Trimethoprim) 100 mg + 20 mg Suspension	86.67	100	3.33
Diazepam 5 mg/ml Injection	76.67	76.67	0
Ferrous salt 30 mg/5 ml Suspension	66.67	83.33	6.67
Gentamycin 40 mg/ml Injection	86.67	73.33	0
Ibuprofen 100 mg/5 ml Syrup	73.33	86.67	0
Isonaized 100 mg Tablet	76.67	0	0
Morphine 10 mg/5 ml Oral Solution	0	0	0
Oral Rehydration Solution Powder to make 1 l	90	100	3.33
Paracetamol 120 mg/5 ml Syrup	73.33	86.67	6.67
Paracetamol 125 mg Suppository	70	93.33	23.33
Penicillin G, Benzanthine n 1.2MIU for Injection	76.67	73.33	0
Phenobarbitone 30 mg Tablet	60	36.67	0
Phenytoin 50 mg Tablet	46.67	40	0
Procaine penicillin 1 MIU Powder for Injection	26.67	13.33	0
Salbutamol puff 100mcg/dose Inhaler	66.67	96.67	3.33
Vitamin A 100,000 IU Capsule	43.33	0	0
Zinc sulfate 20 mg Tablet	90	93.33	0

*MIU* Million international unit, *IU* International unit, *LP* Lowest-priced, *HP* Highest-priced

The average availability for lowest-priced medicines in the public and private sectors were 57.67 and 53.67% respectively. The highest-priced medicines’ average availability in private sectors was found to be 3.87%. When the level of health facility for medicine availability was considered, private pharmacies lead both sectors having 71.6% followed by General Hospitals, 68.39% (Table 3).

**Table 3:** Availability of children essential medicine per study area, sector, and level of health facility in Southern Ethiopia

Study Area	Average Availability of Medicines					
	Public Sector (*n* = 30)	Private Sector (*n* = 30)		**Level of Health Facility**	**Sector**	
	**LP**	**LP**	**HP**		Public Sector (*n* = 30)	57.67
Gurage Zone	60.00	64.44	0.74		1.General Hospital	68.39
Hadiya Zone	58.62	62.22	0.74		2.Primary Hospital	58.62
Halaba Zone	61.38	65.19	6.67		3. Health Center	57.28
Hawasa City	63.45	66.67	2.22		Private Sector (*n* = 30)	53.67
Kembata-Tembaro Zone	59.31	60.74	4.44		4. Pharmacy	71.60
Wolaita Zone	62.07	64.44	4.44		5. Drug Store	62.04

*LP* Lowest-priced, *HP* Highest-priced

### Costs of EMs

Of 27 EMs, 22 which were found in ≥4 public drug outlets sold 1.56 times their IRPs. The MPRs of 11 EMs were higher than 1.5. Free of charge, artesunate 60 mg, coartem 120 mg, isoniazid 100 mg, and vitamin A 100,000 IU were given. There were 23 EMs purchased 2.60 times their IRPs in the private sectors. Pursuant to Gelders S. et al*,* 14 EMs had ≤2.5 MPRs [34]. In more than 4 drug retail outlets in both sectors, 17 EMs have been found.

To estimate the price variation of individual medicines across sectors, the MPR of the 17 LP medicines was determined. Thus, in the public and private sectors, the MPR (25th -75th percentile) was 1.26 and 2.24, respectively. For these EMs, the average lowest-priced (LP) MPR in the public sectors was 1.57 and private sectors 2.54. Of the 17 LP medicines, 11 EMs in the public sector had an MPR ≤ 1.5, indicating that patient prices were appropriate. In the public sector, the most expensive drug marketed at 3.23 times its IRP was the phenobarbitone 30 mg tablet. Only 3 EMs in the private sector had an LP MPR ≥ 2.5, suggesting that they were expensive in the study area relative to the IRPs. Paracetamol 125 mg suppository (MPR = 5.21), the most costly drug in the private sector, was found to be the cheapest in the public sector. (Table 4). In general, with caution, patients in the study region charged appropriate prices for 53 and 59% of 17 EMs in the public and private sectors, respectively.

**Table 4:** Median Price Ratio (the 25th–75th Percentile) of Lowest and Highest Priced Medicines (*n* = 17)

List of medicine available in At least four Medicine outlets	Public LP MPR	Private LP MPR
Amoxicillin 125 mg/5 ml suspension	1.78(1.6–2.28)	2.27(2.23–2.51)
Amoxicillin + Clavulinc acid 156.25 suspension	1.10(0.83–2.17)	2.52(2.33–2.59)
Ampicillin 500 mg powder for injection	1.85(1.16–2.09)	3.01(2.5–3.31)
Ceftriaxone 1 g powder for injection	1.69(1.32–1.86)	2.29(2.17–2.42)
Cloxacillin 125 mg/5 ml suspension	1.04(0.58–1.06)	1.17(0.92–1.29)
Cotrimoxazole 240 mg/5 ml suspension.	1.26(1.17–1.68)	2.09(1.72–1.26)
Diazepam 5 mg/ml injection	1.37(1.03–1.54)	1.65(1.36–1.97)
Ferrous sulfate 30 mg /5 ml	1.02(0.28–1.04)	1.09(0.29–1.3)
Gentamycin 40 mg/ml injection.	1.24(0.74–1.46)	1.75(1.13–2.04)
Ibuprofen 100 mg/5 ml syrup	2.36(1.99–2.68)	3.15(2.68–3.43)
ORS to make 1 L	0.95(0.72–2.06)	3.67(2.45–4.12)
Paracetamol 125 mg suppository	0.65(0.64–0.74)	5.21(3.82–6.79)
Paracetamol 120 mg/5 ml syrup	1.51(0.94–1.95)	2.24(1.80–3.23)
Penicillin G,Benzthine 1.2MIU	1.99(1.49–2.5)	2.78(2.5–3.23)
Phenobarbitone 30 mg tablet	3.23(2.71–3.55)	3.78(2.89–4.85)
Phenytoin 50 mg tablet	1.10(0.5–1.2)	1.19(0.75–2.28)
Salbutamol puff 100mcg/dose inhaler	1.69(1.22–1.83)	1.92(1.83–2.47)

*LP* lowest-priced, *HP* highest-priced, *MPR* median price ratio, *ORS* Oral rehydration salt

### Treatment affordability for prevalent disease with EMs

Assuming all wages go for drug purchasing, Table 5 revealed 81.82% (9/11) and 91.91% (10/11) of standard treatments for prevalent diseases in the public and private sectors with the LP medicines was unaffordable respectively [37]. Purchasing all the studied medicines except ORS and paracetamol 125 mg suppository would take a day and above, requiring 0.2 and 0.5 days’ wages to pay for the recommended dosage, respectively. Acute otitis media treatment with Augmentin 156 mg/5 ml for 10 days was found to be expensive in both sectors. It took a salary of 3.4 days in the public sector and 7.8 days in the private sector for LPGW to afford it.

**Table 5:** Affordability: cost required to cover full course standard treatment against the number of daily wages

Condition to be treated	Drug name, strength, dosage form, dose, route of administration, frequency & treatment duration	Treatment schedule^**24**^ The total amount of drug required to cover the complete treatment regimen^*^	Unit	Average drug Price per Unit (USD)		Number of day’s wage to pay for treatment	
				Public facilities	Private facilities	Public facilities	Private facilities
Mild pneumonia	Amoxicillin 125 mg/5 ml, OS, 30 mg/kg P.O. TID for 7 days	30 mg/kg × 14.5 kg × 3 × 7 days = 9135 mg = 365.4 mL	mL	0.0082	0.0104	3.0	3.8
Severe pneumonia 1. if improves, switch to ‘a’ 2. if doesn’t improve within 48 h, switch to ‘b’	Benzylpenicillin 1.2MIU, Injection, 50,000 units/kg, IM, every 6 h at least for 3 days	50,000 units/kg × 14.5 kg × 4 × 3 days = 8.7MIU ~ 9MIU = 8vial	Vial	0.2620	0.4984	2.1	4.0
	a. Amoxicillin125 mg/5 ml, OS, 30 mg/kg P.O. TID for 7 days	30 mg/kg × 14.5 kg × 3 × 7 days = 9135 mg = 365.4 mL	mL	0.0082	0.0104	3.0	3.8
	b. Ceftriaxone 1 g, Injection, 50 mg/kg/day, IV, for 5 days	50 mg/kg × 14.5 kg × 5 days = 3625 mg ~ 4 g = 4 vial	Vial	0.6726	0.9114	2.7	3.7
Impetigo	Cloxacillin 125 mg/5 ml, Syrup, 100 mg /kg/day P.O. for 7 days	100 mg/kg × 14.5 kg × 7 days = 10,105 mg = 101.5 mL	mL	0.0101	0.0113	1.0	1.2
Diarrhea with some dehydration	ORS to make 1 litter, 1sachet P.O.	some dehydration 75 ml/kg × 14.5 = 1087.5 ml ~ 2sachet	Sachet	0.0808	0.3137	0.2	0.6
Acute otitis media	Amoxicillin 250 mg/ 5 ml, OS, 5 ml, P.O. TID for 10 days	3 × 5 ml × 10 days = 150 ml	mL	0.0080	0.0107	1.2	1.6
	Augmentin 156.25 mg/5 ml, OS, 5 ml, P.O. TID for 10 days	3 × 5 ml × 10 days = 150 ml	mL	0.0223	0.0512	3.4	7.8
Asthma	Salbutamol puff, 1-2puffs, 3–4 times a day	200doses of 1 inhaler	Dose	0.0155	0.0177	3.1	3.6
Pain management	Paracetamol suppository 125 mg, 15 mg/kg, QID for 1 day	15 mg/kg × 14.5 × 4 = 870 mg = 7supp ~ 10supp	Supp	0.0521	0.4173	0.5	4.2
Pain management	Ibuprofen 100 mg/5 ml, Syrup, 10 mg/kg, P.O. PRN	100 mg/5 ml of 100 mL	mL	0.0120	0.0161	1.2	1.6

*OS* Oral Suspension, *P.O* Per oral, *TID* Three times a day, *QID* Four times a day, *PRN* When necessary, *IM* Intramuscular, *IV* Intravenous, *Supp* Suppository, *USD* United States of American Dollar

*Average weight of 5 year age children in Ethiopia =14.5 kg

### General analysis of availability and Price

**Fig. 1:**
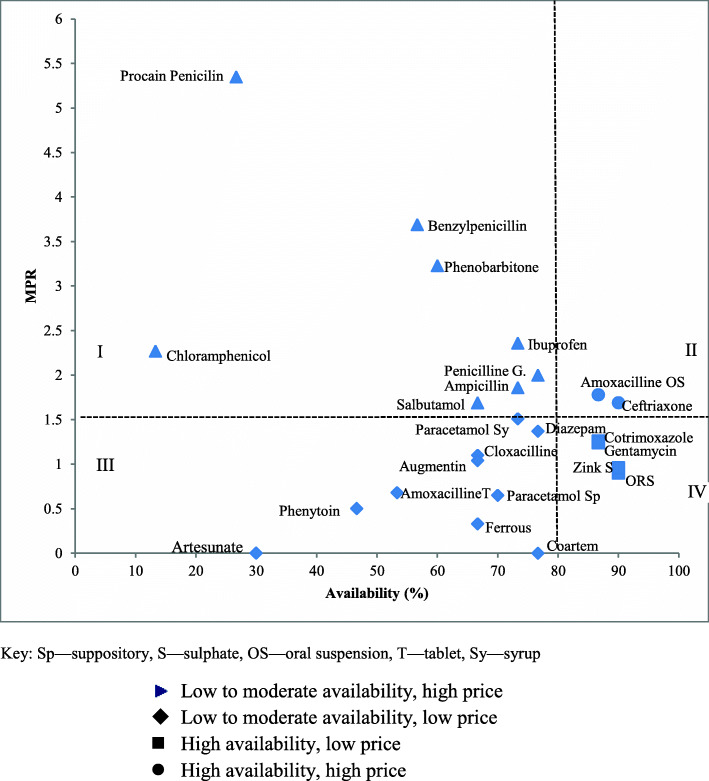
General analysis of medicine availability and retail price in the public sector. On the x-axis, the percent availability for each drug is depicted and on the y-axis, the MPR value is shown

The availability and prices of LP EMs were demonstrated in Fig. 1 for public sectors. The percent availability for each EM was depicted on the x-axis and the MPR value on the y-axis. The Figure was divided into four quadrants, taking into account 80% availability and considering cut-off point MPR = 1.5. Quadrant IV contains EMs with low MPR and high availability. In this segment, only 4 EMs were found. In quadrant I, EMs with high MPR and low availability have been reported, suggesting that patients have trouble accessing and affording them. If there were no alternative medication for infectious diseases, the absence of chloramphenicol in in private sectors (Supplementary Fig. 1) and the high price and low availability shown in Fig. 1 would have made infection control potentially difficult.

## Discussion

The current study utilized Chahal, H.S. et al*,* work to present the cut-off for the EMs availability percent range. Accordingly, 6 and 12 EMs were highly available (> 80%) in the public and private sectors respectively [31]. Private sectors were good at having highly available EMs compared to their encounters. This may be due to their flexible reordering time, and refilling their consumption before stock-out looking at demand trends. Public-sectors are abide by law when and form whom to reorder—they are not permitted to procure simply because of EMs are below certain level. They have to follow stirict rules and wait until their reorder time. Such low availability of overused EMs are usually occurs as a result of poor consumption forecasting and procurement. Since stock-movement in both sectors is not similar, only 5 of these highly available EMs were found. The supply of highly consumable EMs in the public sector will decline as it reachs the store of the HFs before the day of reorder, while refilling is immidiate in private sectors as there is fast stock movemnt. On the other hand, for 3 EMs in the public sector and 6 EMs in the private sector, low availability (< 30%) was reported, with 3 EMs being < 30% in both. Eighteen and 7 EMs in the public and private sectors respectively kept a broader range (30–80%) of availability.

The average availability of LP medicines for children was fairly high in both sectors [31]. It was 57.67% in public sectors and 53.67% in private sectors. However, none of the selected districts’ HFs stocked beclomethasone inhaler, morphine 10 mg/5 ml oral solution, and carbamazepine 100 mg/5 ml suspensions. Regarding the higher availability of medicines in the public sector compared to the private sector, these findings are consistent with the results of a study done by Edao Sado and Alemu Sufa, in the Western part of Ethiopia, for a similar target [27]. Studies like the compiled reports of WHO and Anson A et al results disagree with the current work by finding low average availability of medicines in the public than private sectors [7, 33]^.^

PFSA, the country’s largest source of medicine, is now turning its office work into the field [38]. It helps the clients to engage from medicine selection to rational use. It is trying to have the actual needs of each health institution found in the country. This shift could allow health institutions to increase the availability of EMs.

The average availability of medicines used to treat chronic conditions such as seizure disorders and asthma in children was low (≈42%) [31]. As carbamazepine and beclomethasone (alternative EMs) were totally absent, there was no mitigation for the observed low availability. This is attributed to parents’ inadequate knowledge of diseases and the weak capacity of health facilities to diagnose and manage cases [9]. Appropriate demand definition reports should therefore not be correctly established to acquire adequate supply.

Medicines offered free of charge from the public sectors like artesunate 60 mg and vitamin A were found below 50%. This is because malaria is a seasonal epidemic. Its drug stock usually varies. Only when the need arises, drugs such as artesunate and coartem are procured and refilled free of charge from the source (Regional Health Bureau). Otherwise, the inventory resides in the central store. Regarding vitamin A, the service is mainly provided by health posts and they were also not part of this research. Private sectors do not have much interest in stock because these drugs are dispensed free of charge and their demand is low. When they disregard isoniazid stocking, such lack of interest was assured. In addition, the prescriber’s desire for other alternatives, the negative thinking relating to opioid abuse, and being categorized under the Narcotic and Psychotropic Substance (NPS), caused morphine not to be stocked.

Infectious diseases are known causes of childhood morbidity and mortality [11, 12, 16]. The availability of medicine used to tackle these conditions has to be maintained at the optimum level (≥ 50%). However, the average availability of chloramphenicol 1 g was below the ‘very low’ level [31]. Presence of safe alternative medicines and unwanted effects of chloramphenicol in children caused a decline in demand and supply. Procaine penicillin could not be held in the majority of studied drug sources due to the update of the treatment protocol. The dispersible tablet of augmentin (amoxicillin 125 mg + clavulinic acid 31.25 mg) was found in 2 public and 2 private sectors. As children prefer the form of suspension dosage to the tablet, and due to the price issue it was hardly available. Amoxicillin 250 mg dispersible tablet as a result of low interest/low priority by consumers and benzyl penicillin due to lesser/no stock movement, private sectors showed less willingness to include them in their retails.

Irrational antibiotic use, on the other hand, may decrease the availability of EMs during the study period in the study area. Since they are prescribed for diseases unconfirmed by laboratory diagnosis, such as for viral origin, or prescribed if not required, or the poor controlling system that could not give up obtaining them without a prescription for self-medication could affect the stock [39].

The current study also showed that the overall retail prices of the LP medicines were higher than their IRPs. They were sold at 1.26 times their IRPs in the public sectors and 2.24 times their IRPs in the private sectors. Concerning substantially higher prices in private sectors compared to public sectors, this finding is similar to the studies done by Edao Sado and Alemu Sufa, and Sun X et al [27, 40]. A noticeable price variability between both sectors was common for captured medicine in this study. It is consistent with a study undertaken on the availability, prices, and affordability of essential medicines in Ethiopia, Haiti, and china [27, 31, 40]. Such higher than IRPs prices observed in the studied EMs were attributed to (i) the fact that it is appropriate for the public sector to add up to 40% of procurement cost to the price of each EM, while (ii) the private sector having unsolved issues with PFSA, pointing to its costly sources and merely looking at the demand trend (and/or stock-out pattern at public sector), it may unreasonably add exaggerated sums of money for procurement costs to the price. As well as protection, quality, and effectiveness, the medication price control issue emphasized under the National Drug Policy has not been enforced for several reasons [41]. For certain opportunists, such gaps are expedient environments. For example, paracetamol 125 mg suppository was the cheapest EM in the public sector, but in the private sector, it was the most costly EM. The country is promoting local medicine production instead of price regulation and enforcing licensed medicine stocking and dispensing institutions to contract with PFSA.

Managing commonly prevalent conditions—acute and chronic— with standard treatment protocol using the LP medicines in the region was unaffordable (81.82% in public and 91.91% in private) as they cost a day’s or above wage for the LPGW. This finding agrees with the findings of Edao Sado and Alemu Sufa, and Sun X et al [27, 40]*.* The assumption of the LPGW method for determining the affordability of EMs is that all wages go towards the purchase of medication. For households with an average of 4.6 children, it is not obvious to spend a day’s wage buying medication alone [42]. Low-income earners are likely to spend 93 and 60% of their income on food, housing, transport, utilities, and sport or leisure activities as per Mokaya J et al. and Xu K et al. findings respectively [43, 44]. Accordingly, for healthcare expenses just 7 and 40% of income are left. In the current work, the LPGW requires 0.4 to 2.3 days’ wage (1.33–7.67% income) to afford the cheapest LP medication ORS in the public sector. This would be all right for Mokaya J et al [43]. For an expensive standard treatment of acute otitis media with Augmentin 156.25 mg/5 ml in the private sector, the high income (40%) left is not enough to accommodate as it needed the LPGW’s 19.4 to 110.8 days’ wage (64.67–369.33% income). Thus, almost all the 11 standard treatment options identified in this work were unaffordable. This showed that the government’s target of achieving universal health coverage for its citizens through CBHI and the donor partnership does not seem to improve access because affordability remains an unresolved problem. Not only does the way CBHI exercise impact the accessibility of EMs or the affordability of care, it also deteriorates the entire operation of health institutions, as CBHI financing is handled by those who are least concerned with health and unable to produce the bill on time for the purchase/refill of supplies. Since most people earn less than the specified income at the LPGW, they have either forgo treatment, tried other local healing activities, suspend their basic needs or borrowed.

## Limitation of the study

This study did not assess factors affecting or related to availability, price, and affordability.

## Conclusion

The average availability of EMs for children in this work was fairly good. Public sectors have relatively higher availability than private sectors provided that government-subsidized, free of charge offered and public sectors only allowed to stock medicines were included in the study. However, the average LP MPR for public and private sectors being 1.57 and 2.54 times their IRPs compromises children’s access to EMs respectively. Furthermore, being unaffordability of LP medicines for 81.82 and 90.91% of full-course standard treatments of prevalent conditions in the public and private sectors as they cost a day’s or above wages for the LPGW respectively, lowering childhood morbidity and mortality questionable.

## Supplementary Information

**Additional file 1: Annex I.** Information sheet and consent form. **Annex II.** Medicine price collection data form of public HF and private MOs. **Annex III.** Price in USD and IRP of children essential medicine in public and private sectors. **Annex IV.** Selected diseases from the top ten Prevalent childhood illness in the region to measure affordability for treatment. **Supplementary Figure 1.** General Analysis of Availability and Price for private sector. On the x-axis, the percent availability for each drug is depicted and on the y-axis, the MPR value is shown.

## Data Availability

The datasets used and/or analyzed during the current study available from the first author on reasonable request (email: teffjan99@gmail.com).

## Abbreviations

CEMs: Children essential medicines
EMLc: Essential medicine lists for children
EMs: Essential medicines
ETB: Ethiopian birr
HAI: Health action international
HC: Health center
HFs: Health facilities
HP: Highest-priced
IRPs: International reference prices
LPGW: Lowest-paid government unskilled worker
LP: Lowest-priced
MPR: Median price ratio
MS: Microsoft
SNNP: Southern nations nationalities and peoples
WHO: World health organization
