# Supplementary material for: Availability and affordability of children essential medicines in health facilities of southern nations, nationalities, and people region, Ethiopia: key determinants for access
Source: BMC Public Health. 2021 Apr 13;21:714. doi: 10.1186/s12889-021-10745-5 (PMC8045262; doi:10.1186/s12889-021-10745-5)
Supplement: Supplementary file 1 — Additional file 1: Annex I. Information sheet and consent form. Annex II. Medicine price collection data form of public HF and private MOs. Annex III. Price in USD and IRP of children essential medicine in public and private sectors. Annex IV. Selected diseases from the top ten Prevalent childhood illness in the region to measure affordability for treatment. Supplementary Figure 1. General Analysis of Availability and Price for private sector. On the x-axis, the percent availability for each drug is depicted and on the y-axis, the MPR value is shown. [file 12889_2021_10745_MOESM1_ESM.docx]

**AVAILABILITY AND AFFORDABILITY OF CHILDREN ESSENTIAL MEDICINES IN HEALTH FACILITIES OF SOUTHERN NATIONS, NATIONALITIES, AND PEOPLE REGION, ETHIOPIA: Key Determinants for Access.**

**ADDITIONAL INFORMATION**

# Annex I. Information sheet and consent form

This study is aimed to assess the availability and affordability of essential medicines in SNNPR, ETHIOPIA. To collect data, I, ____________________ invite you to take part in my data collection. If you are willing, you need to understand and sign the consent form. By participating in this research, you may sacrifice your time to provide an important input to show the gap and improve the availability and affordability of essential medicines. The output of the study will have both direct and indirect benefits to you, as you and your families will use the services in the future. You will not be provided any incentive or payment for your good deeds from the researchers except thanking. The data that I collect from your facility be kept private. Any information about you will have a number on it instead of your name and ownership. Only the researcher will know what your number or code is. It will not be shared with or given to anyone except advisor and data collector. You have the full right to refuse from participating. Your refusal will not affect you from providing any kind of health-related services within the community. If you want to know more information, you can contact the following:

Tefera Tadesse(0941005807, Email [teffjan99@gmail.com)/](mailto:teffjan99@gmail.com)/); Habtamu Abuye (0913571974, Email [harohabe@gmail.com)/](mailto:harohabe@gmail.com)/).

**Consent form**

With the due understanding of the above information are you willing to participate in the study?

**Yes** (Proceed with the interview)

**No** (terminate the interview)

Interviewer

Name_________________________________sign______________date__________

Supervisor/researcher remark and signature

Name ___________________________sign__________________date____________

Code____________________________ woreda_________________

# Annex II. Medicine price collection data form of public HF and private MOs

1. Health facilities information

Date: ________________ Study area: --------------------------------------

Name of town/district: ______________________________________

Name of health facilities (optional):____________________________________

Health facility unique data collection ID (mandatory):__________________________

**Type of medicine outlet:**

| Public [PU] | | Private [PR] | | Remark |
| --- | --- | --- | --- | --- |
| Primary | 1 | Licensed Pharmacy | 1 |  |
| Secondary | 2 | Licensed drug store | 2 |  |
| Tertiary | 3 |  |  |  |

**Type of price:**

- Price the patient pays

**Type of data:**

Sample outlet ______ Back-up outlet______ Validation visit _____

Name of manager of the medicine outlet and/or person(s) who provided information (if different from manager):__________________________________________

Name of data collectors: _______________________________ __________

Verification

To be completed by the area supervisor at the end of the day, once data have been verified

Signed: _______________________________ Date: ________________

Code no. -----------

| A | B | C | D | E | F | G | H | I | J |
| --- | --- | --- | --- | --- | --- | --- | --- | --- | --- |
| Medicine name,  Dosage form, strength | Available  Today | Medicine type | Brand product or name | Manufacturer | Target pack size | Pack size found | Price of the pack found | Unit price /4 digits/ | Comment |
| Amoxicillin 125mg/5ml suspension | ▫Yes  ▫No | LP |  |  | 100ml |  |  | /ml |  |
|  |  | HP |  |  | 100ml |  |  | /ml |  |
| Amoxicillin 250mg dispersible tablet | ▫Yes  ▫No | LP |  |  | 21 |  |  | /tab |  |
|  |  | HP |  |  | 21 |  |  | /tab |  |
| Amoxicillin+clavulanic acid125mg+31.25mg/ 5ml suspension | ▫Yes  ▫No | LP |  |  | 100ml |  |  | /ml |  |
|  |  | HP |  |  | 100ml |  |  | /ml |  |
| Amoxicillin/clavulanic acid 125mg+31.25mg/ 5ml dispersible Tablet | ▫Yes  ▫No | LP |  |  | 21 |  |  | /tab |  |
|  |  | HP |  |  | 21 |  |  |  |  |
| Ampicillin 500mg injection | ▫Yes  ▫No | LP |  |  | 1vial |  |  | /vial |  |
|  |  | HP |  |  | 1vial |  |  | /vial |  |
| Artemether+Lumefantrine 20mg + 120mg  dispersible tablet* | ▫Yes  ▫No | LP |  |  | 6*1 |  |  | /tab |  |
|  |  | HP |  |  |  |  |  |  |  |
| Artesunate 60mg injection | ▫Yes  ▫No | LP |  |  | 1vial |  |  | /vial |  |
|  |  | HP |  |  | 1vial |  |  | /vial |  |
| Beclomethasone inhaler 100mcg/dose | Yes  ▫No | LP |  |  | 1inhaler |  |  | /dose |  |
|  |  | HP |  |  | 1inhaler |  |  | /dose |  |
| Benzylpenicillin 600mg = 1 million IU injection | ▫Yes  ▫No | LP |  |  | 1vial |  |  | /vial |  |
|  |  | HP |  |  | 1vial |  |  | /vial |  |
| Carbamazepine100mg/  5ml Suspension | ▫Yes  ▫No | LP |  |  | 100ml |  |  | /ml |  |
|  |  | HP |  |  | 100ml |  |  | /ml |  |
| Ceftriaxone 1g vial for injection | ▫Yes  ▫No | LP |  |  | 1vial |  |  | /vial |  |
|  |  | HP |  |  | 1vial |  |  | /vial |  |
| Chloramphenicol 1g vial for injection | ▫Yes  ▫No | LP |  |  | vial |  |  | /vial |  |
|  |  | HP |  |  |  |  |  |  |  |
| Cloxacillin 125mg/ 5ml suspension | ▫Yes  ▫No | LP |  |  | 100ml |  |  | /vial |  |
|  |  | HP |  |  | 100ml |  |  | /vial |  |
| Cotrimoxazole 200mg + 40mg  Suspension | ▫Yes  ▫No | LP |  |  | 100ml |  |  | /ml |  |
|  |  | HP |  |  | 100ml |  |  | /ml |  |
| Diazepam 5mg/ml  Injection | ▫Yes  ▫No | LP |  |  | 2ml |  |  | /ml |  |
|  |  | HP |  |  | 2ml |  |  | /ml |  |
| Ferrous salt 40mg Fe/5mlsuspension | ▫Yes  ▫No | LP |  |  | 200ml |  |  | /ml |  |
|  |  | HP |  |  | 200ml |  |  | /ml |  |
| Gentamycin  40mg/ml injection | ▫Yes  ▫No | LP |  |  | 2ml vial |  |  | /ml |  |
|  |  | HP |  |  | 2ml vial |  |  | /ml |  |
| Ibuprofen 100mg /5ml syrup | ▫Yes  ▫No | LP |  |  | 100ml |  |  | /ml |  |
|  |  | HP |  |  | 100ml |  |  | /ml |  |
| Isoniazid 100mg *scored tablet | ▫Yes  ▫No | LP |  |  | 56 |  |  | /tab |  |
|  |  | HP |  |  | 56 |  |  | /tab |  |
| Morphine10mg/5ml oral solution | ▫Yes  ▫No | LP |  |  | 100ml |  |  | /ml |  |
|  |  | HP |  |  | 100ml |  |  | /ml |  |
| Oral re-hydration  solution to 1 litter | ▫Yes  ▫No | LP |  |  | 1sachet |  |  | /sachet |  |
|  |  | HP |  |  | 1sachet |  |  | /sachet |  |
| Paracetamol120mg/5ml Suppository | ▫Yes  ▫No | LP |  |  | 100sup |  |  |  |  |
|  |  | HP |  |  | 100sup |  |  |  |  |
| Paracetamol  120mg/5ml syrup | ▫Yes  ▫No | LP |  |  | 100ml |  |  | /ml |  |
|  |  | HP |  |  | 100ml |  |  | /ml |  |
| Penicillin G, Benzanthine 1.2miu | ▫Yes  ▫No | LP |  |  | 1vial |  |  | /vial |  |
|  |  | HP |  |  | 1vial |  |  | /vial |  |
| Phenobarbital 30mg tablet | ▫Yes  ▫No | LP |  |  | 1000 |  |  | /tab |  |
|  |  | HP |  |  | 1000 |  |  | /tab |  |
| Phenytoin 50mg tablet | ▫Yes  ▫No | LP |  |  | 200 |  |  | /tab |  |
|  |  | HP |  |  | 200 |  |  | /tab |  |
| Procaine penicillin  1 gram = 1 million  IU injection | ▫Yes  ▫No | LP |  |  | 1vial |  |  | /vial |  |
|  |  | HP |  |  | 1vial |  |  | /vial |  |
| Salbutamol100mcg/dose Inhaler | ▫Yes  ▫No | LP |  |  | 1inhaler |  |  | /dose |  |
|  |  | HP |  |  | 1inhaler |  |  | /dose |  |
| Vitamin A 100,000IUcapsule | ▫Yes  ▫No | LP |  |  | 50 |  |  | /cap |  |
|  |  | HP |  |  | 50 |  |  | /cap |  |
| Zinc 20mg  dispersible tablet | ▫Yes  ▫No | LP |  |  | 14 |  |  | /tab |  |
|  |  | HP |  |  | 14 |  |  | /tab |  |

Note: * Not assessed in private facilities since both medicines are supplied through the program.

#

# Annex III. Price in USD and IRP of children essential medicine in public and private sectors

| **S.no** | **Name of medicine found at least in 4 outlets** | **Unit** | **Public HF** | **Private MOs** |  |
| --- | --- | --- | --- | --- | --- |
|  |  |  | Unit Price /USD | Unit Price /USD | IRP |
|  | Amoxicillin 125mg/5ml suspension | mL | 0.0082 | 0.0104 | 0.0046 |
|  | Amoxicillin + Clavulinc acid 156.25 suspension | mL | 0.0223 | 0.0512 | 0.0203 |
|  | Ampicillin 500mg powder for injection | Vial | 0.2788 | 0.4536 | 0.1507 |
|  | Ceftriaxone 1gm powder for injection | Vial | 0.6726 | 0.9114 | 0.3980 |
|  | Cloxacillin 125mg/5ml suspension | mL | 0.0101 | 0.0113 | 0.0097 |
|  | Cotrimoxazole 240mg/5ml suspension. | mL | 0.0060 | 0.0100 | 0.0048 |
|  | Diazepam 5mg/ml injection | mL | 0.0796 | 0.0959 | 0.0581 |
|  | Ferrous sulfate 30mg Fe/5ml | mL | 0.0438 | 0.0468 | 0.0429 |
|  | Gentamycin 40mg/ml injection. | mL | 0.0744 | 0.1050 | 0.0600 |
|  | Ibuprofen 100mg/5ml syrup | mL | 0.0120 | 0.0161 | 0.0051 |
|  | ORS to make 1L | Sachet | 0.0808 | 0.3137 | 0.0850 |
|  | Paracetamol 125mg suppository | Supp | 0.0521 | 0.4173 | 0.0801 |
|  | Paracetamol 120mg/5ml syrup | mL | 0.0079 | 0.0116 | 0.0052 |
|  | Penicillin G, Benzthine 1.2MIU | vial | 0.3483 | 0.4865 | 0.1750 |
|  | Phenobarbtone 30mg tablet | Tab | 0.0242 | 0.0284 | 0.0075 |
|  | Phenytoin 50mg tablet | Tab | 0.0385 | 0.0417 | 0.0350 |
|  | Salbutamol puff 100mcg/dose inhaler | Dose | 0.0155 | 0.0177 | 0.0092 |

# Annex IV: Selected diseases from the top ten Prevalent childhood illness in the region to measure affordability for treatment

| **Condition to be treated** | **Drug name, strength & dosage form** |
| --- | --- |
| Mild pneumonia | Amoxicillin of 125mg, 30mg/kg P.O. TID for 7 days |
| Severe pneumonia | Benzylpenicillin of 1MIU, 50,000units/kg every 6hours at least 3days |
|  | Amoxicillin 30mg/kg TID for 7days |
|  | Ceftriaxone 50mg/kg/day for 5days |
| Impetigo | Cloxacillin 100mg /kg/day for 7days |
| Diarrhea with some dehydration | ORS to make 1litter, 1sachet |
| Acute otitis media | Amoxicillin 250mg 5ml TID for 10days |
|  | Augumentin suspension 156mg/5ml TID for 10days |
| Asthma | Salbutamol puff, 1-2puffs 3-4 times a day |
| Pain management | Paracetamol suppository 15mg/kg QID for 1day |
| Pain management | Ibuprofen syrup 10mg/kg |

Supplementary Figure 1: **General Analysis of Availability and Price for private sector**

Key: Sp—suppository, S—sulphate, OS—oral suspension, T—tablet, Sy—syrup

- Low availability, high price
- Low availability, low price
- High availability, low price
- High availability, high price

On the x-axis, the percent availability for each drug is depicted and on the y-axis, the MPR value is shown.

Operational Definition of terms

The following definitions are only for the purpose of this study,

**Access—**is physical and economical accessibility of essential medicine/EM/ in SNNPR public and private health facilities during the period of study.

**Affordability—**refers to the economical accessibility of EM in SNNPR selected health facilities in the period of study in relation to the daily wage of the lowest-paid unskilled national government worker.

**Availability—**refers to physical accessibility of a single dose of medicine/ tab, cap, ml… / in SNNPR health facilities/ private and public/ on the day of visit with the specified strength and dosage forms.

**Child—**a child in this study refers children younger than five year age.

**Health facilities—**includes Hospitals and Health centers having own outpatients dispensing area and licensed private medicine outlets.

**Key tracer essential medicines—**refer to selected tracer medicines in the Ethiopian National Essential Medicines List that must be available at all times in a health service delivery point.

**Lowest priced—**are medicines with the lowest unit price available at each medicine outlet on the day of the survey and identified at individual facilities. If only one type of medicine is found in medicine outlet on the day of visit in the health facilities of SNNPR, it will be considerd as lowest priced medicine.

**Mark up cost—**refers to the amount of profit margin added on procurement price by public and private health facilities in SNNPR.

**Percentile—**the range of values containing the central half of the observations: that is, the range between the 25th and 75th percentiles (the range including the values that are up to 25% higher or down to 25% lower than the median) is called the inter- quartile range.

**Price—**is expressed as a price per unit (e.g. tablet, dose) and was converted in to a median price ratio (MPR) only if the medicine is available in at least four facilities by dividing the median local price by an international reference price (IRP).

- Percentage of medicine outlets which was selected medicine on the day of data collection:

$$Percentage Availability=\frac{Number of Medicine Outlets Actually had the Individual Medicines on the Day}{Total Number of Medicine Outlets}*100\%$$

$$Average Percentage Availability=\frac{Number of Children EMs in Stock on that Day}{Number of children EMs Reviewed}*100\%$$

- The overall availability of essential medicines in facilities was reported as “the average percentage availability of the medicines in the facilities on the day of data collection”.
